# Medical News & Visiting List

**Published:** 1889-12

**Authors:** 


THE BUFFALO LITHIA SPRINGS.—We again invite attention to these excellent springs, as referred to in an advertisement in this Journal, headed *Buffalo Lithia Water*.

The profession everywhere recognize the great value of these waters, especially in kidney and bladder troubles. Dyspepsia, rheumatism, gout, etc. See the advertisement.

